# Clinical features of ischemic stroke in patients with nonvalvular atrial fibrillation combined with intracranial atherosclerotic stenosis

**DOI:** 10.1002/brb3.3036

**Published:** 2023-05-01

**Authors:** Ning Wu, Xinli Wang, Shuai Jia, Xiaomei Cui, Yaozhen Wang, Jian Li, Xiaojun Zhang, Yanqiang Wang

**Affiliations:** ^1^ Department of Neurology II Affiliated Hospital of Weifang Medical University Weifang Shandong China; ^2^ Department of Neurorehabilitation Yidu Central Hospital of Weifang Weifang Shandong China

**Keywords:** clinical characteristic, intracranial atherosclerotic stenosis (ICAS), ischemic stroke, nonvalvular atrial fibrillation (NVAF), pathogenesis, risk factors

## Abstract

**Background:**

Nonvalvular atrial fibrillation (NVAF) and intracranial atherosclerotic stenosis (ICAS) are major causes of ischemic stroke. Relatively few studies have focused on the risk factors and clinical features of ischemic stroke caused by NVAF combined with ICAS.

**Method:**

We retrospectively evaluated NVAF and/or ICAS in patients with acute ischemic stroke admitted within 72 h after stroke. All patients with acute ischemic stroke underwent diffusion‐weighted magnetic resonance imaging (DWI), magnetic resonance angiography (MRA), computed tomography angiography (CTA), and/or digital subtraction angiography (DSA). NVAF was detected by routine electrocardiogram or 24‐h Holter examination, Doppler echocardiography, and contrast echocardiography of the right heart.

**Results:**

Among the 635 enrolled patients, NVAF, ICAS, and NVAF+ICAS were diagnosed in 170 (26.77%), 255 (40.16%), and 210 (33.07%) patients, respectively. Patients in the NVAF+ICAS group were older (*p* < .001), specifically aged ≥75 years (*p* < .001). The admission time of the NVAF+ICAS group was shorter (*p* < .001) than that of the ICAS group. The admission NIHSS score of the NVAF group was higher than that of the NVAF+ICAS group (*p* < .001). HsCRP, NTpro‐BNP, and LEVF levels were significantly different among the three groups (*p* < .001). NVAF+ICAS ischemic stroke occurred mainly in the right hemisphere (52.4%).

**Conclusion:**

NVAF with ICAS ischemic stroke is more likely to occur in older patients. Infarctions occurred mainly in the right cerebral hemisphere. Neurological deficits in NVAF are more severe than those in NVAF combined with ICAS and in simple ICAS ischemic strokes. HsCRP, LEVF, andNTpro‐BNP seem to be closely associated with NVAF+ICAS ischemic stroke.

## Graphical abstract

In this work, we explored the clinical features of ischemic stroke in patients with nonvalvular atrial fibrillation (NVAF) combined with intracranial atherosclerotic stenosis (ICAS). At present, there are few studies on ischemic stroke caused by NVAF combined with ICAS in domestic and foreign researches, and sound scientific evidence is still lacking.

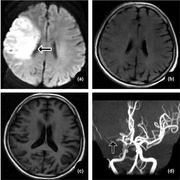

## 1 INTRODUCTION

Ischemic stroke is the main cause of death and disability among adults and has become a major public health concern worldwide (Katan & Luft, 2018). In recent years, China has observed and increased stroke burden. A previous study reported on the prevalence of stroke in China between 2013 and 2019. In most provinces of China, the prevalence of stroke continues to increase annually, and the weighted prevalence of stroke is higher for male sex, older age, and residence in rural and northeast areas (Tu et al., 2022). Patients with cardioembolism usually present with severe neurological deficits and recurrent strokes. Atrial fibrillation (AF), especially nonvalvular atrial fibrillation (NVAF), is associated with more than half of all cardioembolisms and early neurological deterioration (Ogata & Yasaka, 2007). Numerous studies have explored the risk factors and pathogenesis of ischemic stroke due to NVAF (Benbir et al., 2007; Chang et al., 2002; Kim et al., 2015; Matsumoto et al., 2013). However, not all ischemic strokes with NVAF are related to cardioembolism; the occurrence of ischemic stroke in patients share many modifiable and nonmodifiable risk factors, and there are also some important differences in clinical practice (Chang et al., 2002; Sun et al., 2018). Some patients with ischemic stroke with NVAF also present cerebral atherosclerosis (Abolbashari, 2022; Sun et al., 2018). Clinical observations have shown that most patients with cerebral atherosclerosis have intracranial atherosclerotic stenosis (ICAS) in Asia, especially in China (Niu et al., 2014). The interaction mechanism between NVAF and ICAS in the occurrence and development of ischemic stroke remains unclear; therefore, the pathogenesis, treatment options, and prevention strategies for ischemic stroke are complicated by the coexistence of NVAF and ICAS.

Clinical studies have reported high‐risk factors for ischemic strokes caused by NVAF or ICAS, and their overlapping factors include age, hypertension, diabetes, dyslipidemia, homocysteine levels, and metabolic syndrome (Benbir et al., 2007; Wang et al., 2017). However, most of these results indicated a positive correlation between multiple risk factors and ischemic stroke. Only a few studies have reported the differences in the risk factors and related mechanisms of NVAF combined with cerebral atherosclerosis, and sound scientific evidence is still lacking. In this study, we retrospectively analyzed patients with ischemic stroke with first NVAF combined with ICAS and sought to identify the relevant risk factors and mechanisms to provide meaningful references for further prevention and treatment.

## 2 MATERIAL AND METHODS

### 2.1 Patients

**FIGURE 1:**
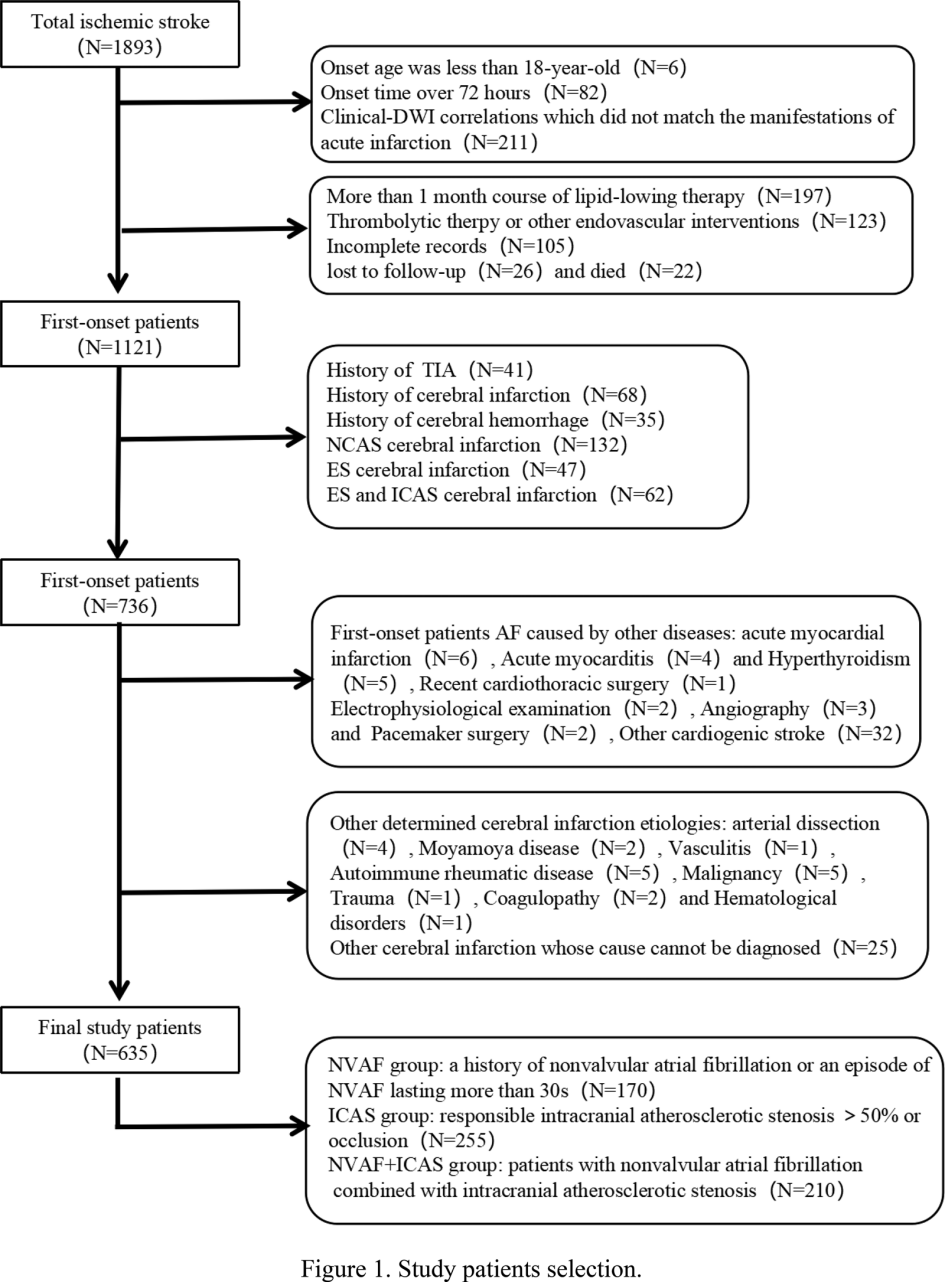
Study patients selection.

We retrospectively studied patients with first‐ever ischemic stroke (IS) (≥18 years old, < 72 h of onset) who were admitted to the Affiliated Hospital of Weifang Medical University from September 2015 to October 2021. Of the 1893 consecutive patients, we selected 635 patients to enroll in this research (Figure 1). The patients underwent diffusion‐weighted magnetic resonance imaging (DWI), magnetic resonance angiography (MRA), computed tomography angiography (CTA), and/or digital subtraction angiography (DSA). All patients had a DWI/apparent dispersion coefficient (ADC) lesion that matched their clinical stroke presentation. NVAF was detected by routine electrocardiogram or 24‐h Holter examination, Doppler echocardiography and contrast echocardiography of the right heart.

Clinical data were collected, including demographics (sex, age, admission NIHSS score, and admission time), and risk factors (hypertension, diabetes, coronary heart disease, hyperlipidemia, smoking history, alcohol consumption), defined according to the previous reports (Men et al., 2013; NCEP, 2002). Clinical data from the following imaging modalities were also collected: magnetic resonance imaging (MRI) with MRA, CTA or DSA, carotid duplex ultrasonography, electrocardiography or 24‐h electrocardiography (Holter), Doppler echocardiography and contrast echocardiography of the right heart (within the next 3 days after stroke). Fasting (no caloric intake for at least 8 h) venous blood samples were collected within 24 h of admission. The laboratory tests included homocysteine (Hcy), high‐sensitivity C‐reactive protein (HsCRP), white blood cell (WBC), platelet count (PLT), red blood cell (RBC), hemoglobin (Hb), glycohemoglobin A1c (HbAlc), uric acid (UA), D‐dimer, fibrinogen (FIB), N‐terminal pro‐B‐type natriuretic peptide (NTpro‐BNP), left ventricular ejection fraction (LEVF), total cholesterol (TC), triglycerides (TG), high‐density lipid cholesterol (HDL‐C), and low‐density lipid cholesterol (LDL‐C).

The exclusion criteria were as follows: (1) clinical‐DWI correlations that did not match the manifestations of acute infarction; (2) prior history of ischemic stroke, transient ischemic attack (TIA), or cerebral hemorrhage; (3) a history of thrombolytic therapy or other endovascular interventions; (4) a course of more than 1 month for lipid‐lowering therapy; (5) noncerebral artery stenosis (NCAS) or extracranial stenosis (ES) or extracranial and intracranial stenosis related ischemic stroke; (6) AF due to reversible causes including acute myocardial infarction, acute myocarditis, untreated hyperthyroidism, or due to treatment examinations including recent cardiothoracic surgery, electrophysiological examination, angiography, and pacemaker surgery; (7) other cardiogenic stroke: potential emboligenic cardiac diseases, recent myocardial infarctions(< 4 weeks), valvular atrial fibrillation, prosthetic valves, dilated cardiomyopathy, rheumatic mitral or aortic valve disease, left atrial appendage thrombosis, left ventricular thrombosis, dyskinetic or akinetic left ventricular aneurysm, atrial myxoma, infective endocarditis, complex atheroma in the ascending aorta or proximal arch; (8) other determined ischemic stroke etiologies: arterial dissection, Moyamoya disease, vasculitis, autoimmune rheumatic disease, malignancy, trauma, coagulopathy, or hematological disorders; (9) other ischemic stroke whose cause cannot be diagnosed; (10) incomplete records and loss to follow‐up.

### 2.2 Clinical data and assessments

Patients with AF were characterized by a history of AF or an episode of AF lasting more than 30 s. Electrocardiography or 24‐h electrocardiography (Holter) showed absolutely irregular RR intervals and no discernible or distinct P waves lasting for at least 30 s (the frequency was between 350 and 600 beats per minute) (Chen et al., 2018). Patients with ICAS with significant intracranial stenosis (those with ≥50% stenosis or occlusion) were characterized by duplex imaging or arteriography according to the methods described in the Warfarin Aspirn for Symptomatic Intracranial Disease Study (Samuels et al., 2000; Warfarin‐Aspirin Symptomatic Intracranial Disease (WASID) Trial Investigators, 2003). The diagnosis of stroke secondary to atherosclerosis cannot be made if duplex or arteriographic studies are normal or show only minimal changes.

Intracranial arteries included the distal (including the cavernous and petrosal segments) internal carotid artery (ICA), middle cerebral artery (MCA), anterior cerebral artery (ACA), posterior cerebral artery (PCA), basilar artery (BA), and distal (including the intradural V4 segment) vertebral artery (VA). Anterior circulation is facilitated by the carotid artery system, which consists of the internal carotid artery and its branches. The posterior circulation is enabled by the vertebrobasilar system, which consists of the vertebral and basilar arteries and their branches. Two neurologists and two neuroradiologists were blinded to the clinical data, MRA data and DWI lesion patterns for evaluation.

### 2.3 Statistical analysis

Data from selected patients were compared among the three groups. Normally distributed numerical variables are expressed as mean ± standard deviation, nonnormally distributed numerical variables are expressed as medians (interquartile ranges), and categorical variables are expressed as percentages. *T*‐test and the Kruskal–Wallis test were used for independent normally and nonnormally distributed continuous variables, respectively, while the *χ* ^2^ or Fisher's exact test was used to analyze the relationships between categorical variables. The risk factors that were significant in the one‐way analysis were determined by multifactor logistic regression, and the odds ratio (OR) and 95% confidence interval (95% CI) were used to estimate the risk of each factor. *p* < .05 was considered statistically significant. All statistical analyses were performed using the SPSS software, version 26.0.

### 2.4 Ethics statement

This study was approved by the local Ethics Committee of the affiliated Hospital of Weifang Medical University. The written or oral informed consent for this study was obtained from the patients or their family members. The research ethics approval number was wyfy‐2022‐ky‐202.

## 3 RESULTS

### 3.1 Baseline characteristics of patients with strokes of different etiologies

As shown in Table 1, a total of 635 patients (average age, 70.70 ± 10.88 years; male, 340 (53.50%); age ≥75 years, 210 (33.07%)) were included in this study. The NVAF+ICAS group had fewer male patients (*p* < .001) than the other groups. Compared with the ICAS group, the NVAF+ICAS group was older (*p* < .001), specifically aged ≥75 years (*p* < .001), and had a shorter admission time (*p* < .001). The admission NIHSS score was higher in the NVAF group than in the NVAF+ICAS group (*p* < .001). The characteristics of patients with a history of smoking, alcohol consumption, diabetes, hypertension, hyperlipidemia, or coronary artery disease did not differ among the three groups (Table 1).

**TABLE 1:** Baseline characteristics of patients with strokes of different etiologies.

Variables	ICAS group (*n* = 255)	NVAF group (*n* = 170)	NVAF+ICAS group (*n* = 210)	*p*	*p*1	*p*2	*p*3
Male, %	160 (62.7)	95 (55.9)	85 (40.5)	.000	.157	.000	.003
Age, years	65(58, 70)	74(70, 81)	74(69, 82)	.000	.000	.000	.213
Age ≥75 years, %	35 (13.7)	75 (44.1)	100 (47.6)	.000	.000	.000	.496
Smoking history, %	85 (33.3)	45 (26.5)	70 (33.3)	.257			
Alcohol consumption, %	65 (25.5)	55 (32.4)	70 (33.3)	.133			
Diabetes, %	85 (33.3)	60 (35.3)	75 (35.7)	.847			
Hypertension, *n*(%)	140 (54.9)	90 (52.9)	110 (52.4)	.849			
Hyperlipidemia, %	40 (15.7)	25 (14.7)	32 (15.2)	.963			
Coronary heart disease, %	45 (17.6)	35 (20.6)	45 (21.4)	.560			
Admission NIHSS score, score	4 (3, 6)	10 (9, 12)	3.5 (3, 5)	.000	.000	.108	.000
Admission time, hour	8 (4, 10)	3.75 (3, 5)	3.5 (2.5, 5)	.000	.000	.000	.072

*p*: comparative three groups; *p*1: NVAF group vs. ICAS group; *p*2: NVAF+ICAS group vs. ICAS group; *p*3: NVAF+ICAS group vs. NVAF group.

NIHSS = NIH stroke scale; NVAF = nonvalvular atrial fibrillation; ICAS = intracranial atherosclerotic stenosis.

### 3.2 Biochemical values for strokes of different etiologies

Table 2 shows the differences in biochemical values among the three groups. HsCRP, NTpro‐BNP, and LEVF levels were significantly different among the three groups (*p* < .001). Hcy, WBC, PLT, RBC, Hb, HbAlc, UA, D‐dimer, FIB, TC, TG, HDL‐C, and LDL‐C levels did not differ among the three groups (Table 2).

**TABLE 2:** Biochemical values for strokes of different etiologies.

Variables	ICAS group (*n* = 255)	NVAF group (*n* = 170)	NVAF+ICAS group (*n* = 210)	*p*	*p*1	*p*2	*p*3
Hcy, μmol/L	14.12 (11.21, 16.16)	14.35 (13.39, 15.34)	14.26 (11.57, 18.22)	.168			
HsCRP, mg/L	5.29 (4.44, 6.35)	10.02 (8.09, 12.81)	11.67 (10.01, 13.21)	.000	.000	.000	.000
WBC, ×10^9^/L	6.11 (5.39, 7.29)	6.46 (5.435, 6.845)	6.14 (5.34, 7.04)	.178			
PLT, ×10^9^/L	214 (189, 255)	218 (187, 266)	219.5 (190, 281)	.177			
RBC, ×10^12^/L	4.28 (3.69, 4.88)	4.21 (4.09, 4.31)	4.26 (3.51, 4.82)	.174			
Hb, g/L	149 (110, 166)	143 (122, 177.75)	145 (140, 153)	.329			
HbAlc, %	6.30 (5.80, 6.80)	6.30 (6.10, 6.70)	6.25 (5.80, 6.70)	.683			
UA, μmol/L	335 (306, 370)	330.5 (311, 367)	344 (318, 365)	.499			
D‐dimer, mg/L	0.96 (0.8, 1.18)	0.97 (0.83, 1.12)	1.005 (0.78, 1.14)	.653			
FIB, g/L	3.15 (2.31, 3.41)	3.11 (2.99, 3.19)	3.11 (2.61, 3.38)	.576			
NTpro‐BNP							
, pg/mL	104.55 (76.54, 307.82)	172.935 (146.2, 218.5)	744.95 (357, 1145)	.000	.000	.000	.000
LEVF, %	62 (57, 67)	53.5 (50, 56)	57 (55, 60)	.000	.000	.000	.000
TC, mmol/L	3.98 (3.39, 4.57)	3.96 (3.64, 4.34)	3.905 (3.74, 4.25)	.643			
TG, mmol/L	1.41 (1.01, 1.72)	1.385 (1.18, 1.55)	1.42 (1.27, 1.51)	.262			
HDL‐C, mmol/L	1.19 (0.97, 1.46)	1.205 (1.08, 1.28)	1.235 (1.06, 1.34)	.924			
LDL‐C, mmol/L	2.99 (2.23, 3.36)	3.08 (2.78, 3.31)	3.015 (2.67, 3.26)	.206			

Hcy = homocysteine; HsCRP = hypersensitive C‐reactive protein; WBC = white blood cell; PLT = platelet count; RBC = red blood cell; Hb = hemoglobin; HbAlc = glycohemoglobin A1c; UA = uric acid; FIB = fibrinogen; NTpro‐BNP = N‐terminal pro‐B‐type natriuretic peptide; LEVF = left ventricular ejection fraction; TC = total cholesterol; TG = triglycerides; HDL‐C = high‐density lipid cholesterol; LDL‐C = low‐density lipid cholesterol; NVAF = nonvalvular atrial fibrillation; ICAS = intracranial atherosclerotic stenosis.

### 3.3 Infarction location in strokes of different etiologies

The locations of the infarct areas are presented in Tables 3 and 4. Of the 635 patients, 390 (61.4%) had anterior circulation stroke, 155 (24.4%) had posterior circulation stroke, and 90 (14.2%) had both. Three groups were more common in terms of anterior circulation; however, the distribution of the infarct hemisphere differed among the groups. ICAS group was common in the left hemisphere (66.7%, 170/255), NVAF group was equal in the both hemisphere (88.24%, 150/170), NVAF+ICAS group was common in the right hemisphere (52.4%, 110/210) (Tables 3 and 4).

**TABLE 3:** Infarction location in stroke of different etiologies.

	Distribution of infarction location				
Group	Anterior circulation stroke	Posterior circulation stroke	Anterior and posterior circulation stroke	χ^2^	*p*
ICAS group, %	155 (60.8)	55 (21.6)	45 (17.6)	7.823	.098
NVAF group, %	105 (61.8)	40 (23.5)	25 (14.7)		
NVAF+ICAS group, %	130 (61.9)	60 (28.6)	20 (9.5)		
Total, %	390 (61.4)	155 (24.4)	90 (14.2)		

**TABLE 4:** Infarction location in stroke of different etiologies.

	Distribution of infarct areas				
Group	Left hemisphere stroke	Right hemisphere stroke	Left and right hemisphere stroke	χ^2^	*p*
ICAS group, %	170 (66.7)	80 (31.4)	5 (2.0)	55.129	.000
NVAF group, %	75 (44.1)	75 (44.1)	20 (11.8)		
NVAF+ICAS group, %	75 (35.7)	110 (52.4)	25 (11.9)		
Total, %	320 (50.4)	265 (41.7)	50 (7.9)		

NVAF = nonvalvular atrial fibrillation; ICAS = intracranial atherosclerotic stenosis.

### 3.4 Multivariate logistic regression analysis of the three groups

**FIGURE 2:**
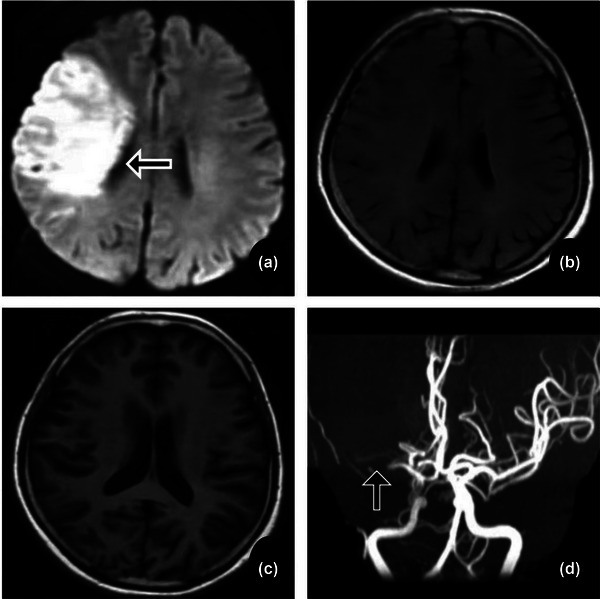
NVAF+ICAS patient. A 68‐year‐old female was admitted 2 h after stroke and she was a NVAF patient. A: DWI, B: T2WI‐FLAIR, C: T1WI, D: MRA. Massive infarct in the right cerebral hemisphere, right MCA occlusion, and right PCA atherosclerotic stenosis.

**FIGURE 3:**
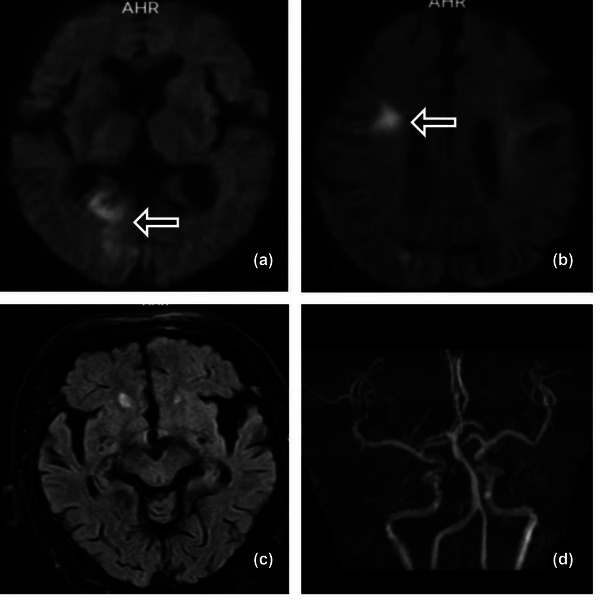
NVAF patient. A 78‐year‐old male was admitted 3.5 h after stroke and he was a NVAF patient. A+B: DWI, C: FLAIR, D: MRA. Multiple infarct in the right cerebral hemisphere. No obvious atherosclerotic stenosis in MRA.

**FIGURE 4:**
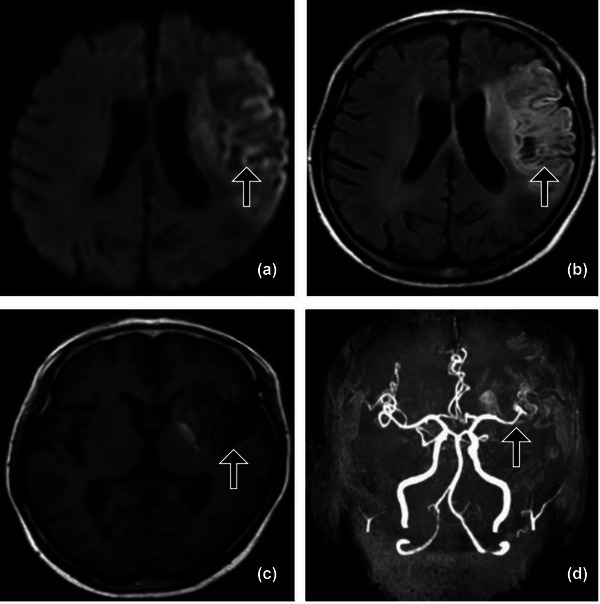
ICAS patient. A 73‐year‐old male was admitted to the hospital 10 h after stroke. A: DWI, B: T2WI‐FLAIR, C: T1WI, D: MRA. Massive infarct in the left cerebral hemisphere, left MCA, and bilateral PCA atherosclerotic stenosis.

Multifactor logistic regression analysis showed that age (OR = 0.809, *p* < .001), age ≥75 years (OR = 11.482, *p* = .007), admission time (OR = 1.587, *p* < .001), HsCRP (OR = 0.397, *p* < .001), NTpro‐BNP (OR = 0.996, *p* < .001), LEVF (OR = 1.123, *p* = .016), and distribution of infarct hemisphere (OR = 27.241, *p* = .035) are independently risk factors between the NVAF+ICAS and ICAS groups. Age ≥75 years (OR = 19.310, *p* = .002), admission NIHSS score (OR = 2.576, *p* < .001), NTpro‐BNP (OR = 0.995, *p* < .001), and LEVF (OR = 0.729, *p* < .001) are independent risk factors between the NVAF+ICAS and NVAF groups (Table 5, Figures 2, 3, 4).

**TABLE 5:** Multivariate logistic regression analysis of the three patients groups (NVAF+ICAS group as a reference).

Group	Variables	OR	*B*	Wald	95% CI	*p*
ICAS	Gender	1.015	0.015	0.001	0.368–2.800	.976
	Age	0.809	–0.212	20.467	0.738–0.887	.000
	Age ≥75 years	11.482	2.441	7.403	1.979–66.627	.007
	Admission NIHSS score	1.089	0.086	0.585	0.875–1.356	.444
	Admission time	1.587	0.462	26.069	1.329–1.894	.000
	HsCRP	0.397	–0.924	58.058	0.313–0.503	.000
	NTpro‐BNP	0.996	–0.004	21.598	0.994–0.998	.000
	LEVF	1.123	–0.116	5.771	1.022–1.235	.016
	Distribution of hemisphere	27.241	3.305	4.445	1.262–588.048	.035
NVAF	Gender	1.243	0.217	0.174	0.448–3.449	.677
	Age	0.924	–0.079	3.335	0.848–1.006	.068
	Age ≥75 years	19.310	2.961	9.630	2.977–125.278	.002
	Admission NIHSS score	2.576	0.946	58.734	2.022–3.281	.000
	Admission time	0.857	–0.154	1.374	0.663–1.109	.241
	HsCRP	0.896	–0.109	3.278	0.796–1.009	.070
	NTpro‐BNP	0.995	–0.005	29.150	0.993–0.997	.000
	LEVF	0.729	–0.316	30.515	0.652–0.816	.000
	Distribution of hemisphere	0.335	–1.095	1.168	0.046–2.438	.280

NIHSS = NIH stroke scale; NVAF = nonvalvular atrial fibrillation; ICAS = intracranial atherosclerotic stenosis.

## 4 DISCUSSION

Many studies have described the pathogenesis and risk factors for ischemic stroke caused by NVAF or ICAS. Nevertheless, few studies have been conducted on ischemic stroke in combination with NVAF and ICAS. These studies only reported the possible characteristics of the coexistence, synergy, dominance, and time correlation of these factors in the occurrence and development of ischemic stroke, without further exploring the risk factors and mechanisms of ischemic stroke combined with their etiology (Kim et al., 2011; Kim et al., 2013; Sanna et al., 2018; Sun et al., 2018). In our study, patients with acute ischemic stroke were divided into the ICAS, NVAF, and NVAF+ICAS groups according to the TOAST classification, imaging examination, and inclusion and exclusion criteria. By comparing the general data and imaging characteristics of the three groups, the risk factors and etiology of NVAF combined with ICAS ischemic stroke were analyzed and explored.

In our study, older patients were more likely to develop NVAF in combination with ICAS ischemic stroke. Advanced age and worse cardiac function have emerged as prethrombotic state markers, which form the basis of cardiac embolism (CE) (Cong & Ma, 2021). The rate of CE in these patients was much higher than that in patients without AF of the same ages (Bos et al., 2012). Older patients are also accompanied with hypertension, diabetes, and hyperlipidemia, which lead to metabolic abnormalities and the formation of unstable atherosclerotic plaques. Therefore, advanced age accelerates the process of NVAF and ICAS simultaneously, leading to ischemic stroke events. We also found that the admission NIHSS score and admission time were important indicators for evaluating the severity of NVAF combined with ICAS ischemic stroke. Collateral circulation did not have sufficient time to compensate when CE occurred. ICAS caused intracranial hypoperfusion and unstable plaques falling off might form new infarcts. Our data demonstrated that the admission NIHSS score was higher in the NVAF group than in the NVAF+ICAS group, which seems inconsistent with these results. We suspected that ICAS is a priority in the infarct mechanism of NVAF+ICAS ischemic stroke. Patients with ischemic stroke with NVAF were prone to have more severe neurological deficits than those with other mechanisms, which shortened the admission time. Further studies are required to investigate the underlying mechanisms.

Inflammation plays a crucial role in the pathogenesis of ischemic stroke. The HsCRP level is positively correlated with the severity of ischemic stroke as an inflammatory factor (Swastini et al., 2019). We also found that HsCRP levels were higher in the NVAF and NVAF+ICAS groups than in the ICAS group. Our data agree with those of previous studies showing that high HsCRP level is a significant pathogenic factor that can be used to estimate the severity and prognosis of ischemic stroke. BNP is an important indicator of impaired cardiac index in this process. After decomposition, released BNP is translated into NTpro‐BNP, which has a longer half‐life, more stable properties, and higher concentration in the blood. It exhibits higher efficiency in predicting stroke than BNP. Meanwhile, ischemic stroke affects the medulla and hypothalamus functioning releasing NTpro‐BNP (Okada et al., 2011; Porzionato et al., 2010). It is worth noting that a study showed that when BNP > 130 pg/mL, patients with AF might be more susceptible to suffering a large atherosclerotic infarction (LAA) (Sakamoto et al., 2019). Our study found that NTpro‐BNP levels in the of NVAF+ICAS group were higher than those in other groups, which suggests that CE and LAA pathogenesis may coexist in NVAF combined with ICAS ischemic stroke. Moreover, high NTpro‐BNP levels are helpful in the identification of NVAF combined with ICAS ischemic stroke. Compared with the NVAF+ICAS and ICAS groups, the NVAF group had significantly lower LEVF values, suggesting that the heart function of a single NVAF ischemic stroke was worse. Mounting evidence indicates that the LEVF is an important index for evaluating cardiac function. AF not only changes the heart structure and remodels atrial systolic function (He et al., 2018), but also activates renin‐angiotensin aldosterone to downregulate LEVF. We hypothesized that NVAF did not play a primary role in the NVAF+ICAS infarct mechanism. These factors, either alone or in combination, affect the occurrence of strokes of different etiologies.

Currently, there is controversy regarding the hemispheric distributions in ICAS ischemic stroke. However, reports have suggested that ICAS with an artery‐to‐artery embolism mechanism principally occurs in the left cerebral hemisphere, the hypoperfusion mechanism occurs in the right cerebral hemisphere, and the infarction volume in the right hemisphere is commonly larger than that in the left (Fink et al., 2002; Naess et al., 2006; Rastogi et al., 2015; Rodriguez Hernandez et al., 2003). We found that the infarction foci were located in the left cerebral hemisphere in most ICAS groups (Figure 4), and this pathogenesis was primarily artery‐to‐artery embolism. It is noteworthy that the different geometries of the aortic arch branches may affect CE laterality. Some studies have shown that NVAF is more common in the right cerebral hemisphere (Figure 3), which is inconsistent with reports that CE ischemic stroke is symmetrically distributed in both hemispheres (Elsaid et al., 2020; Naess et al., 2006; Rastogi et al., 2015; Rodriguez Hernandez et al., 2003). Our results showed that the proportions of the left and right cerebral hemispheres in the NVAF group were equivalent. The proportion of patients with right hemispheric infarction was higher in the NVAF+ICAS group (Figure 2). This may be explained by the pathogenesis of CE and intracranial hypoperfusion. Further prospective studies are needed to understand the laterality of the hemispheric distribution, explore the infarct distribution pattern, and speculate on the pathogenesis of ischemic stroke.

Moreover, Hcy has been reported to be an independent risk factor for ischemic stroke, and can promote the formation of atherosclerosis by mediating the inflammatory response. It was closely correlated with AF. However, there were no significant differences in Hcy levels among the three groups, suggesting that Hcy might be a risk factor. There is no consensus on the relationship between uric acid and ischemic stroke. Some studies believe that uric acid is a major risk factor for ischemic stroke (Verdecchia et al., 2000) and that patients with AF are more susceptible to coexisting with hyperuricemia than the general population (Nyrnes et al., 2014). Nevertheless, other studies (Lee et al., 2009) believed that uric acid was a protective factor with the prognosis of ischemic stroke. Our study did not find a correlation between uric acid and ischemic stroke. This still required to expand the sample size for verification and further analysis.

However, our study has some limitations. First, catheter angiography is considered the standard reference for stenosis detection. MRA is not adequate to replace conventional angiography and is less precise. Second, owning to time and equipment limitations, we did not further explore the subtypes of topographic patterns or the mechanism of NVAF combined with ICAS ischemic stroke. Additionally, bias is inevitable in retrospective studies. First, we enrolled the NVAF and/or ICAS ischemic stroke‐specific populations in our hospital. It was based on data from a single referral center, potentially resulting in selection bias, admission rate bias, or attenuation of statistical power in the analysis of patients with stroke. Thus, our results may not apply to the general stroke population. However, this could have clinical implications, because few studies have attempted to clarify the clinical features of ischemic stroke in patients with NVAF combined with ICAS. Second, this was a retrospective study, the sample size and information collection were relatively insufficient, and there were regional limitations and a long recruitment period, which may have introduced information bias. Finally, although we included risk factors as comprehensively as possible, there may be other confounding factors that we did not consider, which may have confounded the bias.

In conclusion, our findings suggest that ischemic stroke caused by NVAF combined with ICAS is responsible for most strokes in older patients. The right cerebral hemisphere is the main location of stroke lesions. Neurological deficits in the NVAF group were the most severe type of ischemic stroke among the three groups. The HsCRP, LEVF, and NTpro‐BNP biomarkers are of great significance in evaluating patient prognosis.

## CONFLICT OF INTEREST STATEMENT

The authors declare that they have no conflict of interest.

### PEER REVIEW

The peer review history for this article is available at https://publons.com/publon/10.1002/brb3.3036.

## Data Availability

All data are available.
